# Pain Reduction after Short Exposure to Virtual Reality Environments in People with Spinal Cord Injury

**DOI:** 10.3390/ijerph18178923

**Published:** 2021-08-25

**Authors:** David Putrino, Laura Tabacof, Erica Breyman, Jordan Revis, Zulfi Soomro, Divija Chopra, Kathleen Delaney, Anna Smeragliuolo, Mar Cortes

**Affiliations:** 1 Abilities Research Center, Department of Rehabilitation and Human Performance, Icahn School of Medicine at Mount Sinai, New York, NY 10029, USA; laura.tabacof@mountsinai.org (L.T.); erica.breyman@mountsinai.org (E.B.); mar.cortes@mountsinai.org (M.C.); 2 Department of Rehabilitation Medicine, Weill-Cornell Medicine, New York, NY 10065, USA; revisjoj@gmail.com (J.R.); zulfisoomro821@gmail.com (Z.S.); divijachopra@gmail.com (D.C.); kdelaney1@mercymavericks.edu (K.D.); asmeragliuolo@gmail.com (A.S.)

**Keywords:** virtual reality, neuropathic pain, spinal cord injury, neuroplasticity, immersiveness

## Abstract

Emerging literature suggests that virtual reality (VR) may be a viable therapy for neuropathic pain (NP). This pilot study aimed to investigate the immediate effect of VR in reducing NP in people with spinal cord injury (SCI). Eight individuals with chronic NP after SCI were recruited and underwent consecutive exposure to scenery and somatic virtual environments (VE). The numeric rating scale (NRS) was used to assess pain before and after exposure to each VE. The Immersive Tendencies Questionnaire (ITQ) and Presence Questionnaire (UQO-PQ) were used to investigate the interaction between reported pain relief post-intervention with immersion and presence. There was a significant reduction in pain levels (5.1 ± 0.4, mean ± SEM) after short exposure to the scenery (3.1 ± 0.7, *p* = 0.04) and somatic VE (3.0 ± 0.7, *p* = 0.04), with no difference between intervention types (*p* = 0.56). There was a statistically significant negative correlation between the total ITQ score and the change in NRS after the scenery VR intervention (r_s_ = 0.743, *p* = 0.035). PQ scores showed no significant correlation with changes in pain following either intervention type. We found that short-term exposure to VR environments results in a reduction in chronic NP intensity in people with SCI.

## 1. Introduction

Neuropathic pain (NP) affects 40% to 70% of people with spinal cord injury (SCI) and approximately one-third of those affected describe their pain as severe or excruciating [1,2]. It often leads to functional impairments and a decrease in the individual’s quality of life, as about 20% of SCI survivors report that neuropathic pain is more incapacitating than a motor disability [3]. Furthermore, NP often deters people with SCI from completing exercise programs, negatively impacting their rehabilitation process [4].

It has been suggested that persistent NP in people with SCI results from increased depersonalization symptoms originating from a mismatch between the sensory input and the cortical sensorimotor representation of the body [5,6]. Similar mismatch mechanisms have been suggested to underlie phantom pain [7,8,9]. Though NP is mechanistically well understood from the lens of body representation, treatment options for NP are limited and the efficacy of the current recommended treatment options are modest, with many reported side effects and pharmacological intolerances [10,11,12,13].

It is postulated that in people with SCI, the loss of somatosensory drive is thought to generate aberrant nociceptive impulses that are interpreted as pain. Importantly, this results in functional reorganization of the primary somatosensory cortex (SI) and there is accumulating evidence of an association between the degree of this cortical reorganization and the severity of NP [14]. Therefore, strategies aimed at reversing or modulating the somatosensory neural organization may be valuable alternative approaches to treating neuropathic pain.

As an alternative to pharmacological treatment options, the use of virtual illusion in treating neuropathic pain started in 1992 with the use of mirror visual feedback (MVF) in patients with phantom limb pain. The mechanistic theory behind MVF is that it works to reverse maladaptive neuroplastic changes and effects on central regulation [15]. However, MVF only works for unilaterally affected individuals as it requires the ability to move the unaffected limb, limiting its effects on individuals with SCI who typically have bilateral impairments.

Based on the same physiological idea, the use of VR to treat chronic pain started being studied two decades ago and has increased significantly in recent years, showing positive results [16,17,18,19]. In a VR intervention, the observation of goal-directed actions, motor imagery, and action execution may reduce pain by influencing overlapping cortical processes [20,21,22]. The activation of the somatic nervous system, and an increased sense of immersion and presence, could potentially contribute to therapeutic success by regulating somatosensory neuropathic pain and the viewer’s perception of pain [23].

VR has also been shown to reduce pain via distraction among individuals with both acute [24,25] and chronic pain [26]. The underlying analgesic mechanisms of distractive VR remain unclear, though researchers hypothesize that distracting stimuli can decrease perception of pain by downregulating nociceptive neural signaling [27,28]. To date, few studies have investigated the influence of different virtual environments (VEs) on the potential pain-relieving effects of VR. Although there is substantial evidence that VR can result in chronic pain relief [29], the significance of its analgesic effect in SCI-associated neuropathic pain remains unclear [30].

The purpose of this study was to investigate the effect of two different VR protocols on pain intensity in people with SCI. We hypothesized that short-term exposure to a VR protocol would result in an immediate pain reduction in people with SCI and NP and that somatic VEs would produce a greater reduction in pain than scenery VEs. In addition, we also investigated whether participant immersive tendencies and presence influenced the response to the VE.

## 2. Materials and Methods

### 2.1. Study Design and Participants

We performed an individual pilot study with a comparison pre- and post-intervention. Patients underwent two consecutive VR sessions (scenery and somatic) in a randomized order.

Participants were recruited from the Burke Medical Research Institute spinal cord injury database from 2015 to 2016. The inclusion criteria were as follows: (a) presence of chronic neuropathic pain at or below the level of injury for at least 6 months following trauma or disease of the spinal cord, (b) pain intensity of at least 4/10 on the numeric rating scale (NRS) at initial contact, (c) stable pharmacological treatment for at least two weeks prior to study and throughout the trial, and (d) cervical spine control (enough to hold their head in an upright position).

We excluded patients below the age of 18, with severe pain of another origin, with the presence of psychiatric or other neurological disorders, and those with a medical history of head injuries that caused cognitive or visual impairments, severe vertigo, and/or medical instability.

All patients were aware of the purpose of the study. Informed consent was obtained from the patients and the experimental protocol was performed with the full approval of the Burke Rehabilitation Hospital Committee for Human Rights in Research (BRC-527).

### 2.2. Virtual Reality Intervention

**Figure 1:**
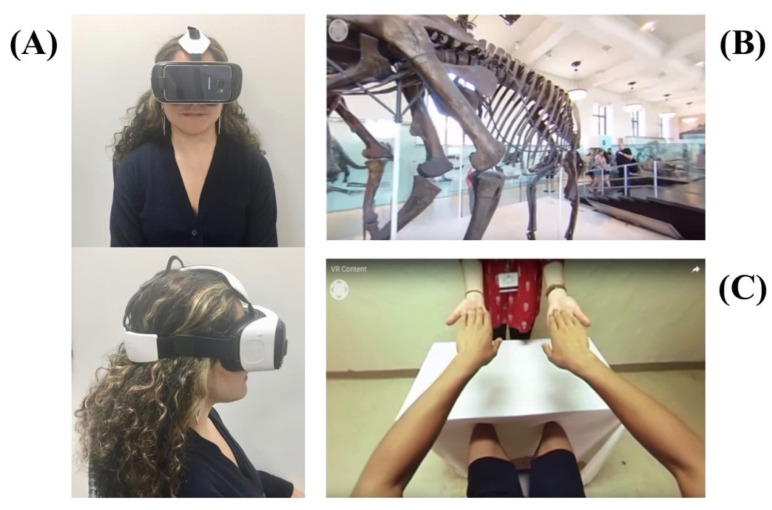
Virtual reality setup for the somatic and scenery virtual environments. (**A**) Samsung Gear virtual reality headset frontal (top) and side view. (**B**) Scenery virtual environment: participants are shown scenes that do not involve somatic interaction with the painful limb (in this case a scene taken from the Museum of Natural History). (**C**) Somatic virtual environment: participants are shown scenes that involve functional movements of the upper and lower limbs. Written informed consent was obtained from the individual for the publication of this image.

Study participants were exposed to two VR environments: scenery, where the participant is exposed to a VE consisting of passive sceneries such as nature experiences; and somatic, where the VE consisted of upper and lower extremity movements (Figure 1). Two types of somatic environments were used, and participants chose the most appropriate environment based upon their self-reported location of their most prominent pain: an upper extremity VE or a lower extremity VE. While viewing the VEs, no specific commands were given, and participants were not encouraged to focus on any particular part or element of the VE. Patients underwent the two consecutive VR sessions (scenery and somatic) in a randomized order. Each VR session was 10 min in duration. The participant was permitted to leave the laboratory and move freely within the campus (in some cases for several hours) and return for the second VR session once the participant reported a return to baseline pain level. 

The VR system consisted of the Samsung Galaxy S3 housed in a Samsung Gear VR headset (Figure 1). Patients were placed in a seated position on a chair or their personal wheelchair, with the ability to look around freely. Patients were instructed to report any discomfort and given the possibility to ask to stop the intervention at any time.

### 2.3. Clinical Assessments and Outcome Measures

The primary outcome for this study was change in neuropathic pain measured through the Numeric Rating Scale (NRS). The NRS scale consists of asking the participant to rate their pain from 0 to 10 (11-point scale), with 0 equal to “no pain” and 10 equal to “worst possible pain”. The NRS provides a valid, reliable, and easy to administer score [31]. NRS pain scores were assessed at baseline and immediately after each VR intervention (scenery and somatic). Participants were also requested to give as many details on the pain characteristics as possible. Reported symptoms of discomfort, dizziness, or nausea were also noted.

The Immersive Tendencies Questionnaire (ITQ) [32] was developed to measure the capability or tendency of individuals to be involved or immersed in a VE. It consists of 18 questions divided into 4 subcategories: focus, implication, emotion, and game, with responses provided according to a 7-point scale ranging from “never” to “often”. The UQO-PQ, the Cyberpsychology Lab version of the presence questionnaire [33], was designed to measure the degree to which individuals experience presence in a VE. The UQO-PQ consists of 5 questions, each rated on a 0 to 100 percent scale. Both questionnaires were completed by each participant at the end of the VR intervention. 

### 2.4. Data Analysis

Post-intervention NRS pain scores were evaluated for significant differences in comparison to baseline using Wilcoxon signed-rank tests. Three datasets were tested for significant differences: (1) Baseline NRS and post-scenery NRS to evaluate whether the scenery VE significantly reduced the participants’ pain score. (2) Baseline NRS and post-somatic NRS to evaluate whether the somatic VE significantly reduced the participant’s pain score. (3) Post-scenery changes in NRS and post-somatic change in NRS to evaluate whether there was a significant difference in the pain-relieving effects of the somatic VE compared with the scenery VE. Additionally, we performed a Spearman correlation to evaluate whether the ITQ and PQ scores correlated with a change in pain following the scenery and somatic VEs.

## 3. Results

### 3.1. Participant Characteristics

Eight people with chronic neuropathic pain after SCI were recruited (four female; 55 ± 3 years, mean ± SEM) for this research study. Five participants presented with cervical lesions, two with thoracic, and one with lumbar (Table 1). The average time since injury was 13 ± 4 years and the average baseline pain levels were scored as 5.1 ± 0.4 on the NRS (Table 2).

### 3.2. Effects of VR on Pain

**Figure 2:**
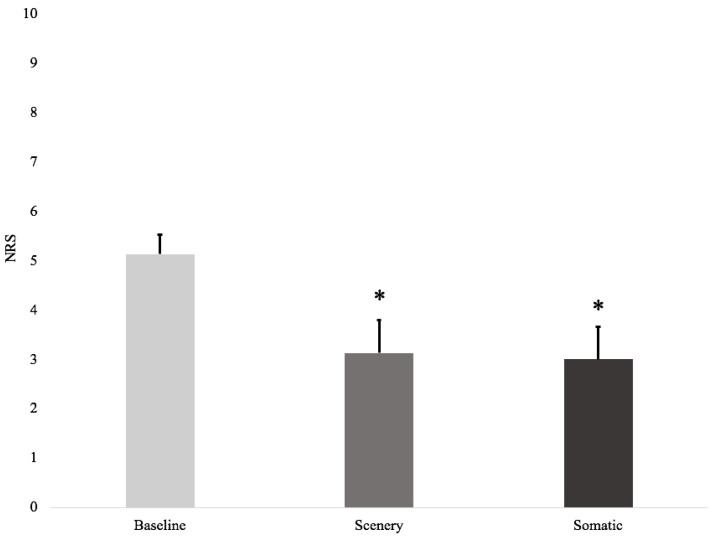
Numeric Rating Scale (NRS) pain scores at baseline before the intervention, post-scenery virtual environment, and post-somatic virtual environment. There was a significant reduction in NRS pain scores after the scenery and somatic virtual environments compared to the baseline. * indicates statistical significance (*p* < 0.05).

There was a significant reduction in relation to baseline pain levels (5.1 ± 0.4, mean ± SEM) after short exposure to the scenery (3.1 ± 0.7, *p* = 0.04) and somatic VE (3.0 ± 0.7, *p* = 0.04). There was no significant difference between intervention types regarding a change in pain scores (*p* = 0.56), see Figure 2. 

### 3.3. VR, Immersion, and Presence

A Spearman correlation analysis revealed a statistically significant negative correlation between total ITQ score and the change in pain after the scenery VR intervention (r_s_ = 0.743, *p* = 0.035). This suggests that the greater the ITQ score, meaning the more immersed one is, the greater the decrease in pain. No significant correlation was found between the total ITQ score and the change in pain after the somatic VR intervention (r_s_ = 0.663, *p* = 0.073). Regarding the subcategories, there was only a statistically significant negative correlation between the focus subcategory of ITQ and the change in pain after the somatic (r_s_ = 0.805, *p* = 0.016) and scenery VR intervention (r_s_ = 0.776, *p* = 0.024). PQ scores showed no significant correlation with changes in pain post-scenery and post-somatic VR interventions.

## 4. Discussion

Our pilot study showed a significant reduction in pain levels following short-term exposure to a virtual environment in people with neuropathic after SCI. However, no significant difference in the magnitude of NRS reduction was observed between scenery and somatic interventions.

VR has been utilized as a pain management tool among individuals with acute [34,35,36], chronic [17], neuropathic [30,37,38,39], and phantom limb pain [40,41], with almost unanimously positive results. VR therapy is also emerging as a promising alternative treatment for NP in people with SCI [30]. Our results showed an average pain NRS decrease of 2.00 (39%) post-scenery and 2.13 (42%) post-somatic VR intervention, which are comparable to effect sizes seen in other studies investigating the utility of VR for chronic pain relief [14,22,42]. Thus, the significant reductions in NRS pain scores compared to baseline after both the scenery and somatic VR interventions that were observed in this pilot trial appear to be consistent with the existing literature surrounding VR and chronic pain. 

Among other alternative pain interventions such as hypnosis, immersion is known to play an important role in governing the responsiveness to the intervention. For instance, a participant’s hypnotizability, meaning how able the participant is to enter a hypnotic state and be immersed in it, is known to influence the efficacy of the intervention [43]. Immersive tendencies and immersion in VR have been studied in numerous domains to determine their effect on other rehabilitation health outcomes such as mobility, postural stability, and functional task performance [44]. However, to our knowledge, the relationship between immersive tendencies and the pain-relieving effects of VR has not been investigated. Our novel finding of a significant negative correlation between immersive tendencies (rated by ITQ) and the change in pain after the scenery VR intervention suggests that the therapeutic response to VR intervention is influenced by the immersive tendencies of the viewer. Furthermore, the significant negative correlation between the focus subcategory of ITQ and the change in pain after the somatic and scenery VR interventions suggests that the participant’s state of mental alertness, capability to concentrate on pleasurable activities, and ability to remove distractions [33] influences the extent to which VR can produce a pain-relieving effect. The mechanisms behind these correlations remain unknown and should be further investigated.

Presence, unlike immersion, did not show a significant correlation with reduction in NRS post-somatic and -scenery VE exposure. The lack of correlation between participant-reported presence and efficacy of a VE has been observed previously, and one hypothesis to support this finding is that presence in a VE is a different quality to engagement in that same VE [45]. Stated another way, a VR user can be aware that they are experiencing a low level of presence in a particular VE, but still be highly engaged due to strong immersive tendencies. This theory is consistent with our findings.

Additionally, both somatic and scenery VEs resulted in a pain reduction among the participants. Though it was initially hypothesized that a somatic VE would result in greater pain reductions than a scenery VE, we observed no significant difference between interventions. The observed reductions in reported pain may be the result of purely distractive mechanisms underlying VR’s analgesic effects [46]. Distraction has certainly been reported as a positive coping strategy for chronic pain patients [47,48]. The observed pain reductions may also be due to underlying mechanistic differences between the somatic and scenery VEs. The mechanisms of distractive VR analgesia rely on the rapid recruitment of brain neuronal networks that activate inhibitory circuits, meaning diverting attention away from pain may reduce available pain-processing resources, thereby resulting in pain reductions [46,49] Pain relief after exposure to a somatic VE may be the result of long-term neuroplastic changes in the sensorimotor regions of the brain, which can lead to complete or partial recovery of both sensory and motor functions [50]. Therefore, a somatic VE would take several sessions, perhaps weeks or months, to induce an effect that is mediated by neuroplasticity [50]. Unfortunately, observation over this timeframe was beyond the scope of the present, pilot study.

In addition, the placebo effect could have also caused similar pain reductions between the somatic and scenery VE. Though placebo-induced analgesia is not as well established for neuropathic pain as it is for nociceptive and idiopathic pain, the placebo effect has been shown to increase the effectiveness of care in neurorehabilitation programs [51]. There is evidence to suggest that placebo- and opioid-induced analgesia share the same neuronal network [52]. Furthermore, higher baseline pain levels were previously correlated to higher placebo-induced analgesia [53], a finding consistent with our results (Table 2). In order to exclude the possibility of pain relief due to placebo or other confounding factors, a control group would need to be incorporated into the study design. However, establishing a sham VR protocol that controls for the possibility of pain reduction due to both distractive and neuroplastic mechanisms poses a methodological challenge for the field that has not yet been comprehensively addressed.

### Study Limitations and Future Directions

This pilot study is limited by the small number of participants, the short duration of the VE, and the lack of a placebo control group. Moreover, the duration of the beneficial effects is unknown due to the lack of a follow-up assessment. Future research will aim to deepen the understanding of the observed correlation between therapeutic response and immersive tendencies by using higher quality VEs alongside more participants with a greater range of immersive tendencies. Understanding this feature with greater granularity may allow for a more personalized approach to prescription of VR-based pain therapies.

Future directions should also include deployment of VR interventions for other populations with similar neural mechanisms of chronic pain (i.e., traumatic brain injury and stroke).

## 5. Conclusions

Our pilot study suggests that short-term exposure to VR environments (somatic or scenery) has the potential to significantly decrease chronic neuropathic pain intensity in people with chronic neuropathic pain. In addition, we found that immersive tendencies, specifically focus, are correlated with neuropathic pain reductions. Future research needs to be conducted in order to confirm these findings and better understand the generalizability and scalability of this innovative approach to treating neuropathic pain. Furthermore, the incorporation of a larger patient population, a control group, randomization, and follow-ups into our study design can allow us to better understand the mechanisms behind the trends observed.

## Figures and Tables

**Table 1:** Participant characteristics. Participant’s sex, age, level of injury, time since injury in years, and American Spinal Injury Association (ASIA) Neurological Classification of Spinal Cord Injury.

ID	Age	Level of Injury	Time Since Injury (Years)	Injury	ASIA
1	56	T4	23	Non-Traumatic	D
2	45	C2	1	Traumatic	D
3	44	C6	22	Traumatic	B
4	50	C5	36	Traumatic	B
5	65	L4	5	Non-Traumatic	D
6	58	C5	3	Traumatic	C
7	71	T10	6	Non-Traumatic	C
8	51	C4	9	Traumatic	C
Mean	55	-	13	-	-
SEM	3	-	4	-	-

**Table 2:** Participant’s pain, immersion, and presence scores. Participant’s baseline and post-intervention scores were recorded.

	NRS			ITQ	UQO-PQ	
ID	Baseline	Post-Scenery	Post-Somatic	Total	Scenery	Somatic
1	4	4	4	55	30	13
2	5	5	5	74	20	8
3	7	5	4	69	38	35
4	5	5	5	68	51	52
5	4	2	2	40	45	13
6	6	2	1	35	44	63
7	4	2	3	55	88	35
8	6	0	0	37	55	40
Mean	5.1	3.1	3.0	54.1	46.4	32.4
SEM	0.4	0.7	0.7	5.5	7.2	7.0

## Data Availability

The data presented in this study are available on request from the corresponding author. The data are not publicly available due to institutional ethics committee restrictions regarding patient privacy.
